# The State and Nursing Education

**Published:** 1921-03-19

**Authors:** 


### THE MATRONS' AND SISTERS' DEPARTMENT.

### THE STATE AND NURSING EDUCATION.

No part of the functions of the General Nursing Council has awakened more interest in nursing circles than that which relates to setting the standard of knowledge which should prevail in the profession. If any misgivings have been felt on this subject, we think they will be dissipated on studying the very able syllabus which has now been forwarded to the chairmen of all hospitals and Poor-law infirmaries, with the request that it may be handed to the matron for her consideration. It is not put forth as definitive. On the contrary, matrons of training-schools and representatives of nursing organisations are invited to attend informal conferences to be held at the rooms of the Royal Society of Medicine, 1 Wimpole Street, at 11 A.M. and 2.30 P.M., on April 28, for the purpose of considering (*a*) matters relating to general training, and (*b*) reciprocal training.

The first thing which will arrest attention in the syllabus is the sharp dividing-line between the first year's training and that of the second and third years. This distinction rests on the observed fact which few matrons or sister-tutors are likely to dispute that so far as real grasp of essential knowledge is concerned the teaching of the first year must be repeated and reinforced or it remains for ever nebulous and of slight account. The first year is the vestibule. In it a multitude of new subjects must perforce be introduced and pass in review before the awakening mind of the probationer. Nothing can be seen in its proper relations to nursing as a whole. Accordingly, we find in this suggested first year's course an outline of many important subjects grouped under the headings of (1) anatomy and physiology; (2) elementary science, including hygiene, sanitation, bacteriology; (3) nursing, theoretical and practical; (4) food values and invalid cooking; (5) first aid. The earlier sections can be adapted for a preliminary training-school course, the nursing sections being necessarily curtailed or postponed until the student begins her ward work. Under the heading of elementary science the nurse is introduced from the very outset of her career to problems affecting the public health and sanitation. This is a most important innovation in the usual scheme of training and the council justify it by the need "to counteract the limited vision sometimes acquired in sick wards." They believe these subjects should be early introduced to the nurse in her training and kept in full view throughout its continuance.

For the second and third years the syllabus is grouped under six main sections—(1) anatomy; (2) physiology; (3) elementary science; (4) gynæcology; (5) surgery; (6) medicine. Each of these sections comprises ten headings or series, and it is suggested they might suitably form a course of lectures on each given subject upon which the pupil would eventually be examined. In general it may be said that there is no attempt to force on the schools an unreal uniformity. Wide latitude is allowed in many directions. Public health and sanitation, for instance, may be introduced under hygiene for elementary treatment or included in a course of chemistry or elementary science. The Council attaches great importance to the lectures and teaching given by fully-trained nurses, *i.e.*, matrons, sister-tutors, ward sisters, etc., and to giving revision classes to pupils with personal supervision of the pupil nurse in all stages of her training and study.

The most vital part of the syllabus (for everyone knows how many nurses succeed in evading instruction under the most admirable syllabus) is the appended "Nurse's Chart." This chart, or something like it, is in use, to name but the pioneers, both at the Middlesex and St. Thomas's Hospitals, and is a valuable *résumé* of the points in practical treatment of the sick which it is imperative all nurses should fully master. The subjects are grouped according to the nature of the work undertaken in each ward, surgical, medical, children's, maternity and gynæcology, or other specialty. Domestic ward management comes first; and this heading includes such advanced details as cost and management of stores, for the points given by no means imply consecutive order in the instruction given, but are dealt with as opportunity offers in the course of the training. On first teaching the points enumerated, the sister of the ward puts a mark in the column descriptive of her ward; one stroke / signifies that the nurse has been shown but is not proficient in the detail; a X means that the nurse has been taught and is proficient. The sister who places the X inserts her initials in the right-hand column. This Chart accompanies the probationer through the entire course, being deposited with the matron at the end of the nurse's work in each ward. By its use the deadly danger of the probationer missing some essential part of her training is completely obviated.

In conclusion, managers will appreciate the remark of the Council that hospital economy as well as ward management should receive more care than appears to be the case in general at present, and that special emphasis should be laid upon economy in the use of hospital property.

